# Effects of Atmospheric Cold Plasma Treatment on the Storage Quality and Chlorophyll Metabolism of Postharvest Tomato

**DOI:** 10.3390/foods11244088

**Published:** 2022-12-17

**Authors:** Sitong Jia, Na Zhang, Haipeng Ji, Xiaojun Zhang, Chenghu Dong, Jinze Yu, Shijie Yan, Cunkun Chen, Liya Liang

**Affiliations:** 1 College of Food Science and Biological Engineering, Tianjin Agricultural University, Tianjin 300384, China; 2 National Engineering Technology Research Center for Preservation of Agriculture Products, Key Laboratory of Storage of Agricultural Products, Ministry of Agriculture and Rural Affairs, Tianjin Key Laboratory of Postharvest Physiology and Storage of Agricultural Products, Tianjin 300384, China; 3 College of Food Science and Nutritional Engineering, China Agricultural University, Beijing 100083, China

**Keywords:** atmospheric cold plasma, tomatoes, chlorophyll metabolism, storage

## Abstract

Atmospheric cold plasma (ACP) is a potential green preservation technology, but its preservation mechanism is still unclear, and the effects of different plasma intensities on postharvest tomatoes are little studied. In this study, the effects of different ACP treatments (0 kV, 40 kV, 60 kV, and 80 kV) on the sensory quality, physiological indexes, key enzyme activities, and gene expression related to the chlorophyll metabolism of postharvest tomatoes were investigated during the storage time. The results showed that compared with the control group, the tomatoes in the plasma treatment group had a higher hardness and total soluble solid (TSS) and titratable acid (TA) contents, a lower respiratory intensity and weight loss rate, a higher brightness, and a lower red transformation rate, especially in the 60 kV treatment group. In addition, chlorophyll degradation, carotenoid accumulation, and chlorophyllase and pheophorbide a mono-oxygenase (PAO) enzyme activities in the postharvest tomatoes were inhibited in the 60 kV treatment group, and the expressions of three key genes related to chlorophyll metabolism, chlorophyll (*CLH1*), pheophytinase (*PPH*), and red chlorophyll catabolic reductase (*RCCR*) were down-regulated. The results of the correlation analysis also confirmed that the enzyme activity and gene expression of the chlorophyll metabolism were regulated by the ACP treatment, aiming to maintain the greenness of postharvest tomatoes.

## 1. Introduction

Tomatoes are popular across the world because of their unique flavor, bright color, and many antioxidants, such as lycopene, carotenoids, and chlorophyll [1,2]. However, the chlorophyll content and physical quality of postharvest tomatoes are decreased due to their high degree of moisture and respiration with the extension of the storage time, which results in the tomatoes turning red and softening, which induces economic losses [3]. Although the traditional chemical preservatives can prolong the storage period of postharvest tomatoes, they have no significant effect in inhibiting tomatoes from turning red, and the residue of chemical preservatives is a serious food safety problem [4].

Atmospheric cold plasma (ACP) is a kind of unstable ionized gas, which contains active substances with bacteriostasis. Additionally, ACP treatment is a potential green preservation technology because of it is less destructive and has no residues [5]. Min et al. [6] reported that the microbes on the surfaces of tomatoes treated with ACP grew slowly, and the sensory quality was well maintained. Zhou et al. [7] found that the microbial growth rate on the surface of cantaloupe was limited by ACP treatment. Similar results were found in studies of blueberries [8], red currants [9], strawberries [10], apples [11], and mulberry [12]. At present, most of the studies mainly focus on the relationship between the bacteriostatic effect and the physiological quality of postharvest fruits and vegetables treated with ACP, and there are few studies related to the effect of ACP treatment on postharvest tomatoes.

The degradation of chlorophyll and the accumulation of carotenoids are signs of the process through which tomatoes ripen and turn red. Studies have shown that the degradation of chlorophyll is closely related to the combined regulation of the chlorophyll enzyme, pheophorbide a mono-oxygenase (PAO), and the chlorophyll enzyme gene, *PAO* gene, chlorophyll hydrolase gene (*PPH*), and red chlorophyll catabolism reductase gene (*RCCR*) [13,14,15]. Zhao et al. [16] found that the slowing of chlorophyll degradation in cherry stems was due to the inhibition of *PAO* and *RCCR* gene expression. Additionally, the downregulation of *PPH* and *PAO* expression slowed the decrease in chlorophyll in the “Comice” pear [17]. In addition, studies have also shown that ACP treatment can induce the type II decomposition of chlorophyll in kiwifruit, resulting in a decrease in the content of chlorophyll A [18]. Baier et al. [19] found that the chlorophyll fluorescence imaging of cucumber was affected by cold plasma treatment. Our study also found that plasma treatment had a positive effect on the green maintenance of postharvest tomatoes, but the effects of ACP treatment on the chlorophyll metabolism and related genes in tomatoes have hardly been studied. The purpose of this study was to explore the effects of different intensities of ACP plasma treatment on the postharvest tomato storage quality and to clarify the most suitable ACP treatment intensity and the influence of the ACP treatment on chlorophyll metabolism in postharvest tomato fruits. This study can provide a basis for the application of ACP preservation technology to postharvest tomatoes.

## 2. Materials and Methods

### 2.1. Tomato Preparation

The green ripening tomato variety used in the trial was Aomei, which was harvested in June 2021 from Baodi, Tianjin, China. Tomatoes in the green fruit stage in shock absorption packaging were immediately shipped to the National Agricultural Products Preservation Engineering Technology Research Center (Tianjin, China). The tomatoes were screened and randomly grouped into 12 portions per group, and 20 fruits were used for each measurement.

### 2.2. Plasma Device and Treatment

The ACP generator was produced by the National Agricultural Products Preservation Engineering Technology Research Center (Tianjin, China) and consisted of a high-voltage power supply and a dual-dielectric discharge module. The power system voltage range was 0–100 kV. The working gas used during the test was air. The tomatoes were treated with ACP at 0 kV, 40 kV, 60 kV, and 80 kV for 5 min and then stored in cold storage at 10 ± 0.5 °C, and the samples were taken in sequence at 0 day, 7 day, 14 day, 21 day, 28 day, and 35 day for observation, with 0 day as the initial value of the observed index.

### 2.3. Firmness

The firmness of the tomatoes was measured using a texture analyzer (TA. XT plus, Stable Micro Systems Ltd., Surrey, Godalming, UK) with a probe diameter of 3 mm and a penetration depth of 1 cm, and 3 points were measured for each fruit, and the results were expressed in kg/cm^2^.

### 2.4. Respiration Intensity

The method of Chen et al. [20] was used to measure the respiration of the tomatoes and was modified slightly. The tomatoes were placed in sealed cans for 3 h, and then the CO_2_ content in the cans was measured using an O_2_/CO_2_ gas analyzer (Check Point, Shanghai Jinchuan Mechatronics Technology Co., Ltd., Shanghai, China). The results were expressed as mg·kg^−1^ h ^−1^.

### 2.5. Weight Loss

The weight loss of the tomatoes was measured with an electronic balance (SENSSUN Shanghai Jinghai Instrument Co., Ltd., Shanghai, China), and the results were expressed as a percentage.

### 2.6. Color

Three points equidistant from the equatorial part of the tomatoes were selected for color detection using a color difference meter (3nh, Guangzhou Ruifeng Experimental Equipment Co., Ltd., Guangzhou, China), in which the values of L*, a*, and b* represented the lightness, redness, and yellowness of the tomatoes, respectively.

### 2.7. Total Soluble Solids (TSS)

The TSS content of the tomatoes was measured according to the method of Wu et al. [21], and the results were expressed as the mass fraction (%).

### 2.8. Titratable Acids (TA)

The method of Ali et al. [22] was used to measure the TA content of the tomatoes, and the results were expressed as the malic acid conversion factor (0.067).

### 2.9. Chlorophylls and Carotenoids

The chlorophyll and carotenoid contents of the tomatoes were detected using the method of Cao et al. with a slight modification [23]. The tomato peel (3 g) was accurately weighed into a mortar and ground until the tissue turned white, and then the absorbance was measured at the wavelengths of 665 nm, 649 nm, and 470 nm using a UV spectrophotometer (TU-1810, Beijing Pu-Analysis General Instrument Co., Ltd., Beijing, China).

### 2.10. Chlorophyllase, Pheophorbide a Mono-Oxygenase

The activities of the chlorophyllase and pheophorbide a mono-oxygenase were determined using a plant chlorophyllase ELISA kit (Shanghai Jianglai Biotechnology Co., Ltd., Shanghai, China). U indicates the magnitude of the enzyme activity. Under the optimal conditions (25 °C), the number of enzymes required to catalyze 1 micromole (μmol) of the substrate into product per minute is defined as one unit of activity.

### 2.11. Expression of the Key Enzyme Genes of Chlorophyll Metabolism

RNA extraction was performed on the tomatoes according to the Trizol method of Wang et al. [24], and reverse transcription was performed with an amount of 1 μg of RNA. Based on the mRNA sequences of the tomato genes (*GADPH*, *PAO*, *PPH*, *CLH1*, *CLH2*, *CLH3*, *CLH4*, *RCCR*) in the GeneBank, the internal reference gene was selected as *GAPDH*, and the primer sequences (5′-3′) used are shown in Table 1. All the genes were subjected to real-time fluorescence quantitative PCR (CFX96 quantitative PCR instrument, Biole Life Medical Products (Shanghai) Co., Ltd., Shanghai, China), and the relative RNA expression was calculated by the 2^−ΔΔCt^ method.

### 2.12. Statistical Analysis of the Results

The measurements were repeated three times for each treatment. The data obtained from the experiments were expressed as the mean ± standard deviation (SD), using Origin 2017 (Origin Lab, Northampton, MA, USA) for the graphing and statistical analysis and IBM SPSS Statistics (version 22.0, Chicago, IL, USA) for the statistical analysis. ANOVA with Tukey’s test was used to analyze the significance of the data with the confidence level of *p* ≤ 0.05. Pearson correlations were also analyzed using IBM SPSS Statistics (version 22.0, Chicago, IL, USA) at the level of *p* < 0.05 and *p* < 0.01 for significant and relatively significant correlations, respectively.

## 3. Results and Discussion

### 3.1. Effects of Different Intensities of the ACP Treatment on the Respiration Intensity, Firmness, TSS Content, TA Content, and Weight Loss Rate of Tomatoes

**Figure 1:**
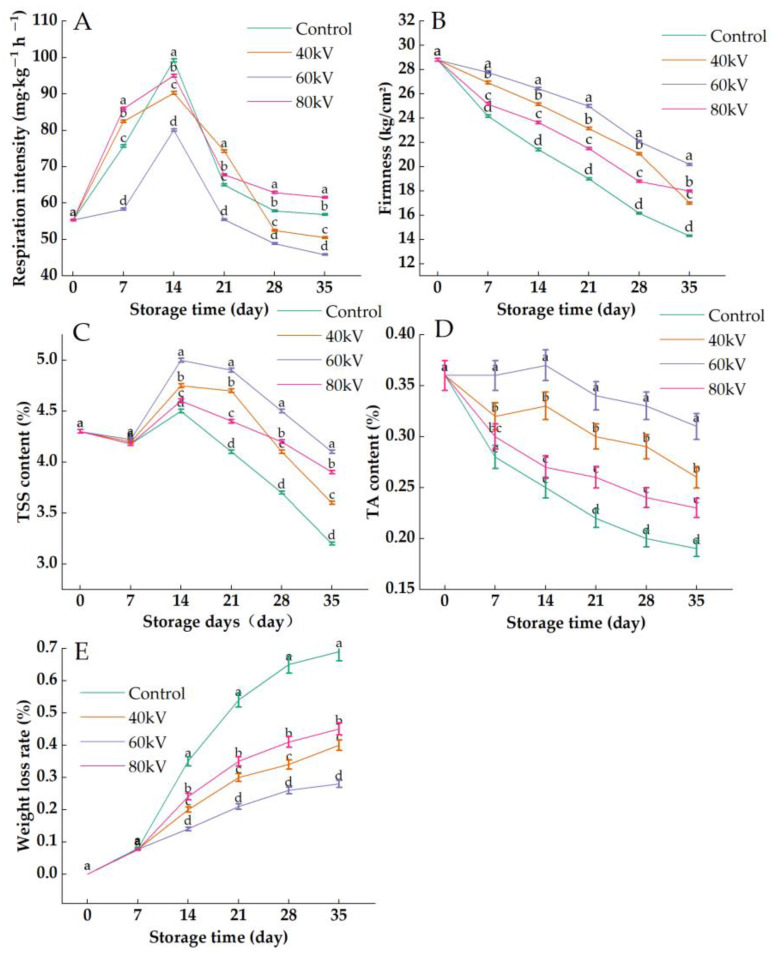
Effects of different intensities of ACP treatment on the respiration intensity, firmness, TSS content, TA content, and weight loss rate of tomato: (**A**) respiration intensity; (**B**) firmness; (**C**) TSS content; (**D**) TA content; (**E**) weight loss rate. Bars labeled with different small letters (a–d) indicate significant differences among different ACP treatments at the same storage time (*p* ≤ 0.05).

The respiratory intensity of the tomatoes during storage is shown in Figure 1A. The respiratory intensity first increased and then decreased among the control and treatment groups and reached its peak after 14 d of storage. The respiratory intensity of the 60 kV treatment group was significantly lower than that of the 40 kV and 80 kV treatment groups and the control group during the whole storage period (*p* < 0.05), indicating that the respiration of the postharvest tomatoes was inhibited by the 60 kV plasma treatment. Misra et al. [10] also found that the respiratory intensity of postharvest strawberries was reduced in the ACP treatment group. The reason for the higher respiratory intensity in the 80 kV treatment group may be the fact that the 80 kV treatment produced more active substances and created an environment higher in reactive oxygen species compared with the 40 kV and 60 kV treatments. Zhang et al. found that high-intensity ACP treatment produced more active substances [25]. As shown in Figure 1B, the firmness of the tomatoes decreased with the prolongation of the storage time, and the firmness of the control group was significantly lower than that of the treatment group (*p* < 0.05). In addition, the hardness of the 60 kV treatment group was significantly higher than that of the 40 kV and 80 kV treatment groups, which may be because the 60 kV treatment group had a low respiration intensity and low consumption of pectin and other substances. Zhou et al. [7] also found that the hardness of cantaloupe after cold plasma treatment was maintained at a high level compared with the control group. The total soluble solids (TSS) and titratable acid (TA) content are important indicators for predicting the quality of postharvest tomatoes [26]. As shown in Figure 1C,D, the TSS content of the tomatoes showed an upward trend in the early storage period and a downward trend after 14 d, while the TA content showed a downward trend over the whole storage time. This may be due to the degradation of the macromolecular carbohydrates that are stored in tomatoes into soluble sugars and organic substances with the increase in respiration [27]. The TSS and TA contents of the control group were significantly lower than those of the treatment group, except for the 7 d sample (*p* < 0.05), which suggested that the plasma treatment had a positive effect on the accumulation of TSS and TA in the postharvest tomatoes. Additionally, the TSS and TA contents were more abundant in the 60 kV treatment group compared to the 40 kV and 80 kV treatment groups (*p* < 0.05), which may be because the 60 kV treatment suppressed the respiration and maintained lower metabolic levels. On the other hand, the consumption of TSS and TA by postharvest tomatoes was accelerated at a higher level of reactive oxygen species (ROS) stress [28], which may explain why the TSS and TA contents in the 80 kV treatment group were lower than those in the 60 kV treatment group [29]. Dong et al. [30] also found that the TSS content in blueberries after cold plasma treatment increased by 1.5 times compared with the control group. The decrease in the TA content of kiwifruit was slowed down by cold plasma treatment [18]. Li et al. [31] also discovered that the TA content of fresh-cut strawberries decreased slowly after ACP treatment.

As shown in Figure 1E, the weight loss rate of the postharvest tomatoes increased gradually with the prolongation of the storage time, but it was similar between the control group and treatment group during the first 7 day. This may be due to the low respiration intensity of the tomatoes and the slow consumption of substances during the early storage time. After the respiratory peak appeared, the weight loss rate of the control group was significantly higher than that of the treatment groups (*p* < 0.05), and the weight loss of the 60 kV treatment group was the lowest, which was consistent with the previous results regarding the TA and TSS contents. Chen et al. [32] also concluded that the weight loss rate of fresh-cut pears was significantly lower than that of the control group after cold plasma treatment.

### 3.2. Effects of ACP Treatment of Different Intensities on the Tomato Color Difference

**Figure 2:**
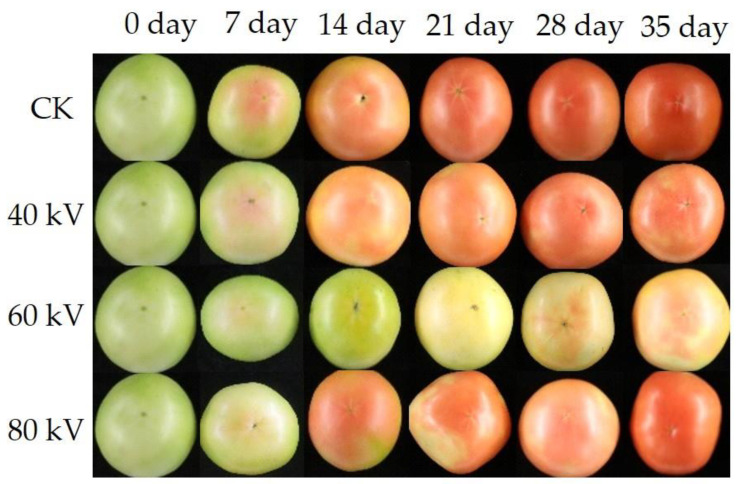
Effects of ACP treatment of different intensities on the apparent changes in the tomatoes.

The color of tomatoes can intuitively reflect their quality. As shown in Figure 2, the redness of the tomatoes was inhibited in treatment groups compared with the control group during the storage time, especially in the 60 kV treatment group. Additionally, as shown in Table 2, the L*, a*, and b* represent the degrees of brightness, green/red, and blue/yellow of the sample, respectively. The L* showed a downward trend in both the control and treatment groups during the whole storage time. However, the L* value of the control group was lower than that of the treatment groups, especially the 60 kV treatment group during storage (*p* < 0.05), which indicated that the ACP treatment could maintain the brightness of the tomato skin. However, the L* value of the 80 kV group was significantly lower than that of the control group in the early stage of storage (*p* < 0.05), which might be because higher levels of ACP can damage the tomato epidermis [33]. Feng et al. [34] also found that the L* value of cherry tomatoes treated with high-pressure argon was higher compared with the control group. The a* value of the tomatoes increased in both the control and treatment groups during storage, which suggested that the tomatoes gradually turn red as the storage period extends. However, the a* value of the 60 kV treatment group was significantly lower than that of the other treatment groups and the control group (*p* < 0.05). The turn red rate of the tomatoes in the control group reached 80%, and the turn red rate of the tomatoes in the 60 kV treatment group was 40% at the end of the storage period. This suggested that an ACP treatment of 60 kV may be beneficial in maintaining the green color of postharvest tomatoes, indicating that chlorophyll metabolism may be regulated through ACP treatment and that the effects of ACP at different intensities are different [35,36,37]. The b* value of each group increased in the first 14 d and then decreased during the storage period. There was no significant difference in the b* value between the treatment groups during storage (*p* > 0.05). This indicated that ACP treatment does not affect the yellowness of tomatoes. To summarize, ACP treatment can inhibit tomatoes from turning red and maintain the tomato skin brightness, and the 60 kV treatment group showed the best effect.

### 3.3. Effects of Different Intensities of ACP Treatment on the Chlorophyll Content, Carotenoid Content, Chlorophyllase Activity, and Pheophorbide a Mono-Oxygenase Activity of Tomato

**Figure 3:**
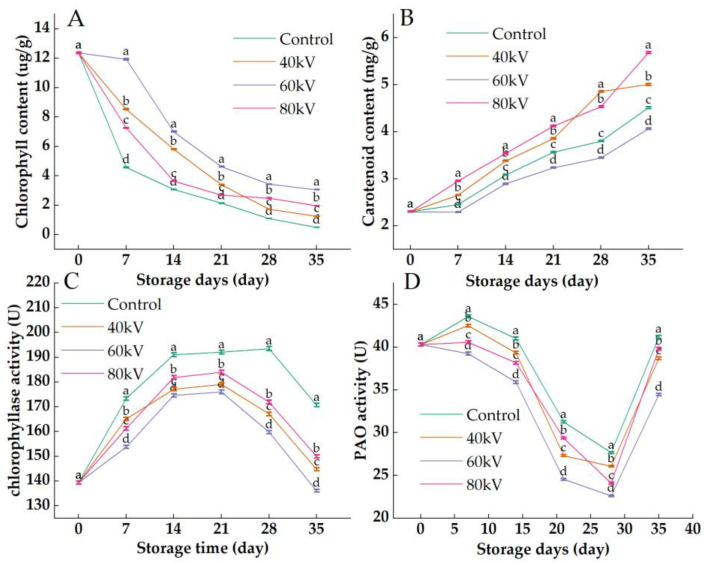
Effects of different intensities of ACP treatment on the chlorophyll content, carotenoid content, chlorophyllase content, and pheophorbide a mono-oxygenase content in tomato: (**A**) chlorophyll content; (**B**) carotenoid content; (**C**) chlorophyllase activity; (**D**) pheophorbide a mono-oxygenase activity. Bars labeled with different small letters (a–d) indicate significant differences among different ACP treatments at the same storage time (*p* ≤ 0.05).

Chlorophyll is gradually degraded into dephytochlorophyll under the effects of chlorophyllase and chlorophyll oxidase (PAO), which induces the appearance of coloring pigments such as carotenoids and the redness of tomatoes. The carotenoids in tomatoes have been found to accumulate less in the early stage of the process of turning red and accumulate rapidly in the later stage of turning red [38,39]. As shown in Figure 3A, the chlorophyll content in the tomatoes showed a downward trend during storage, and the chlorophyll content of the control group was significantly lower than that of the treatment group (*p* < 0.05), which indicated that the plasma treatment delayed the reduction in chlorophyll in the postharvest tomatoes. Additionally, the chlorophyll content of the 60 kV treatment group was significantly higher than that of the 40 kV and 80 kV treatment groups (*p* < 0.05) during storage. Lu et al. [40] reported that the treatment of tomatoes with UV-C could significantly slow down the degradation rate of chlorophyll in the tomatoes and delay the reddening of the tomatoes. The changes in the carotenoid content of the tomatoes are shown in Figure 3B. The carotenoid content of the tomatoes increased during the storage time in the control and treatment groups. However, the contents of carotenoids in the 40 kV and 80 kV treatment groups were significantly higher than those of the control and 60 kV treatment groups, and the carotenoid content in the 60 kV treatment group was significantly lower than that in the control group (*p* < 0.05). It may be that the large amount of oxidizing active substances produced by the 80 kV treatment induced oxidative stress in the postharvest tomatoes, thus inducing the increase in the carotenoid content to neutralize the reactive oxygen species. Chlorophyllase is a rate-limiting enzyme in the chlorophyll metabolic decomposition pathway. As shown in Figure 3C, the activity of chlorophyllase in the tomatoes gradually increased first and then declined rapidly after reaching the peak after 21 d, which indicated that the decomposition of the chlorophyll was accelerated by the increasing activity of chlorophyllase in the postharvest tomatoes in the early stage of redness, and that the large-scale decomposition of the chlorophyll in the tomatoes may result in a relative decrease in chlorophyllase activity at the end of the storage time. Additionally, the activity of chlorophyllase in the control group was always significantly higher than that in the treatment group (*p* < 0.05). In addition, the chlorophyllase activity of the 60 kV treatment group was significantly lower than that of the other treatment groups (*p* < 0.05) during storage. Pheophytin an oxidase (PAO) was another important enzyme participating in the degradation of chlorophyll by interacting with chlorophyll genes [41,42]. As shown in Figure 3D, The PAO activity in the tomatoes showed a downward trend in both the control group and treatment groups during the first 28 d of storage. The PAO activity in the control group was significantly higher than that in the treatment group (*p* < 0.05) during storage, especially in the 60 kV treatment group. Rodoni et al. [43] found that the chlorophyll content in barley leaves decreased with the increase in PAO activity. Wu et al. [44] also concluded that chlorophyll degradation was inhibited in broccoli treated with melatonin by the reduction in the PAO activity. Wang et al. [45] also found that reducing the PAO enzyme activity in broccoli could alleviate the decomposition of chlorophyll. These results suggested that the activity of chlorophyllase and PAO was reduced in the postharvest tomatoes treated with ACP, thus slowing down the degradation rate of the chlorophyll in the tomatoes. This was also confirmed by the changes in the a* value. These results revealed that the different intensities of ACP treatment had different effects on the activity of the enzymes related to chlorophyll metabolism in the postharvest tomatoes, which might be related to the level of reactive oxygen species stress induced by ACP [46,47]. In addition, the 60 kV ACP treatment has the best effect in inhibiting the redness of postharvest tomatoes.

### 3.4. Effects of Different Intensities of ACP Treatment on the Expression of Chlorophyll-Metabolism-Related Genes

**Figure 4:**
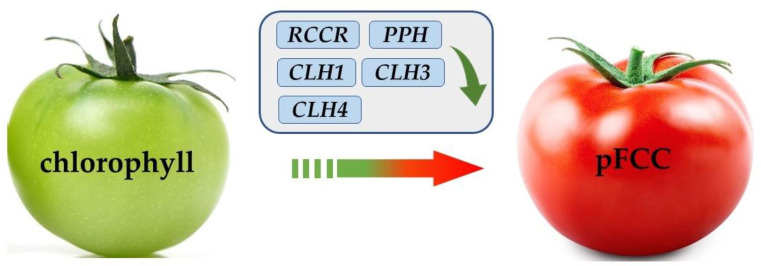
Mechanism of chlorophyll metabolism.

**Figure 5:**
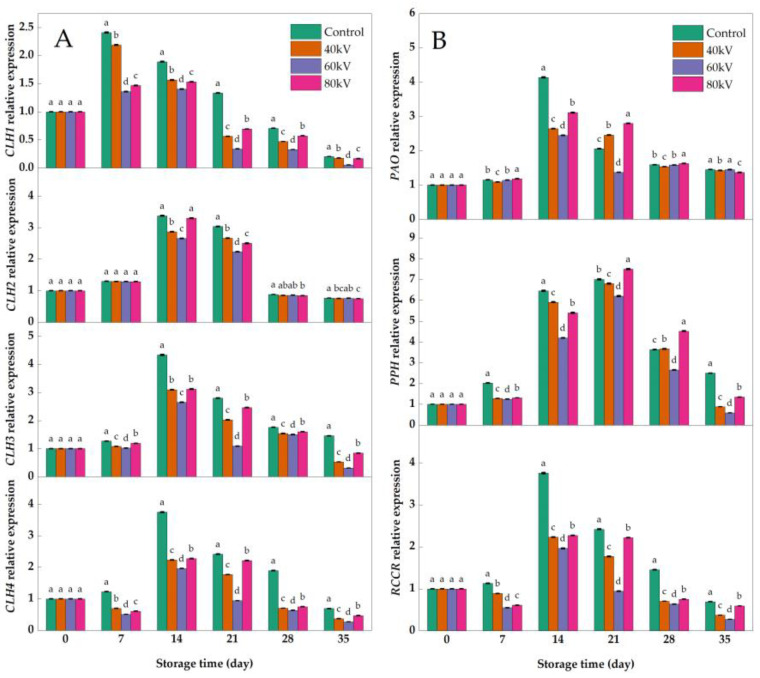
Effects of different intensities of ACP treatment on the relative expression of tomato chlorophyll catabolic genes *CLHS*, *PAO*, *PPH*, and *RCCR*: (**A**) *CLHS* relative expression; (**B**) *PAO*, *PPH*, and *RCCR* relative expression. Bars labeled with different small letters (a–d) indicate significant differences among different ACP treatments at the same storage time (*p* ≤ 0.05).

As shown in Figure 4, the chlorophyll metabolism of tomatoes is divided into primary fluorescent chlorophyll decomposition metabolites (pFCC), which, mainly, are jointly regulated by the pheophytinase gene (*PPH*), chlorophyll enzyme gene (*CLHs*), chlorophyll oxidase gene (*PAO*), and red chlorophyll catabolic reductase gene (*RCCR*), inducing the disappearance of the green color and the appearance of red color in tomatoes [48]. The expression of the *CLHs*, *PAO*, *PPH*, and *RCCR* genes in the postharvest tomatoes was studied between the treatment groups and the control group. As shown in Figure 5A, the expression of the *CLH2* gene was not significantly different between the treatment group and the control group, except for after 14 d and 21 d of storage (*p* > 0.05), while the expression of the *CLH1*, *CLH3*, and *CLH4* genes in the tomatoes was significantly higher in the control group than in the treatment group during storage (*p* < 0.05), which suggested that the *CLH1*, *CLH3*, and *CLH4* genes may be the target *CLH* family genes regulated by the ACP treatment in the postharvest tomatoes. The relative gene expression levels of the *CLH1*, *CLH3*, and *CLH4* genes in the 60 kV treatment group were significantly lower than those of the other treatment groups (*p* < 0.05), which was consistent with the results for the chlorophyll content, carotenoid content, and chlorophyllase activity. The gene relative expression levels of the 40 kV and 80 kV treatment groups were higher than that of the 60 kV treatment group, which indicated that the regulation of the genes related to chlorophyll metabolism in the postharvest tomatoes was related to the ACP intensity [49,50]. Wang et al. [51] also reported that the gene expression of *CLH1* was inhibited and that the yellowing of broccoli was delayed significantly. Zhang et al. [52] also pointed out that melatonin significantly downregulated *CLH1* expression in apple leaves, thereby slowing down the degradation of chlorophyll. Studies also found that the degradation of chlorophyll in pears was delayed by reducing the relative expression of *CLH* family genes [53]. The relative expression levels of *PAO*, *PPH*, and *RCCR* are shown in Figure 5B. The expression levels of the three genes all showed a trend of first increasing and then decreasing in both the control and treatment groups during storage. However, the relative expression levels of the *PPH* and *RCCR* genes in the 60 kV treatment group were significantly lower than those in the other treatment groups and the control group (*p* < 0.05). Compared with the control group, the relative expression of the *PAO* gene in the treatment groups was not significantly different (*p* > 0.05) during storage, except at the 14 d and 21 d timepoints. The results indicated that the relative expressions of *PPH* and *RCCR* were significantly downregulated in the tomatoes treated with 60 kV ACP, while there was no significant effect on the expression of *PAO*. Lv et al. [54] reported that 1-MCP could delay chlorophyll degradation by downregulating the relative expression of the *PPH* and *RCCR* genes in apples. Du et al. [55] also pointed out that the relative expression of *PPH* and *RCCR* in green pepper was downregulated, thus slowing down the degradation of chlorophyll and maintaining the green color. Overall, the relative expressions of *CLH1*, *CLH3*, *CLH4*, *PPH*, and *RCCR* were downregulated in the postharvest tomatoes treated with ACP, thereby reducing the degradation of chlorophyll in the tomatoes and delaying the reddening of the tomatoes. Additionally, the 60 kV treatment group had the most observable effects among the three treatment groups.

**Figure 6:**
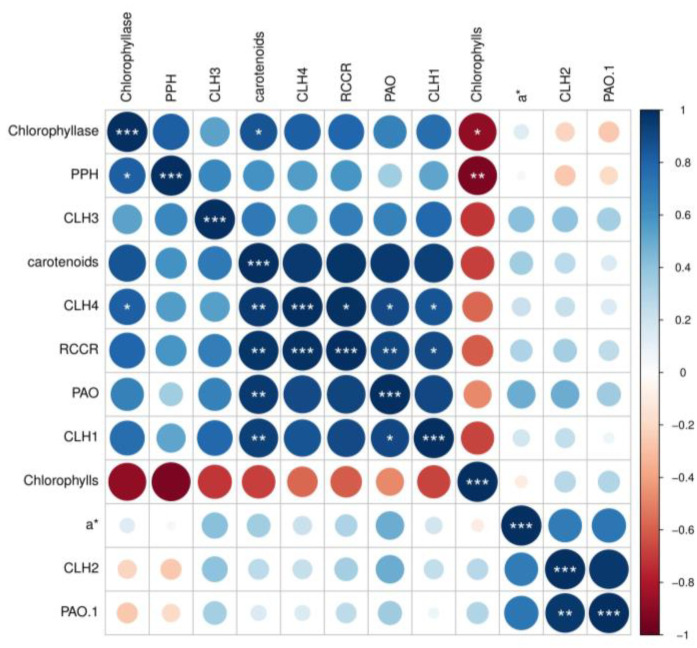
The Pearson correlation coefficient was calculated based on the biochemical parameters and the expression levels of the genes related to the metabolism of chlorophyll in postharvest tomatoes. The heat map was generated based on the correlation of the difference between the 60 kV treatment group and the control group, where *PAO.1* is the *PAO* gene, * is *p* ≤ 0.05, ** is *p* ≤ 0.01, and *** is *p* ≤ 0.001.

Correlation analysis was performed to further investigate the effect of the 60 kV treatment on the chlorophyll metabolism of the postharvest tomatoes during the storage time. As demonstrated in Figure 6, the accumulation of chlorophyll was negatively correlated with the expression of the *PPH*, *CLH1*, *CLH3*, *CLH4*, and *RCCR* genes and the activity of the chlorophyllase, while a positive correlation between the carotenoid content and the levels of the *PPH*, *CLH1*, *CLH3*, *CLH4*, and *RCCR* genes and the activity of chlorophyllase were investigated in the postharvest tomatoes during storage time. These results indicate that ACP of an appropriate intensity can induce changes in the chlorophyll metabolism so as to maintain the green color of postharvest tomatoes. In addition, the chlorophyll content, carotenoid content, chlorophyllase, *PAO* activities, and *CLH1*, *CLH3*, *CLH4*, *PPH*, and *RCCR* genes were clustered together, while the distance between the expression levels of the *CLH2* gene and *PAO* gene was close, which suggested that the *PAO* and *CLH* genes may not be the important genes that ACP regulated, and these results were consistent with the previous conclusions.

## 4. Conclusions

The results showed that the respiration intensity and weight loss of the postharvest tomatoes were inhibited in the ACP treatment groups, and the ACP treatment maintained a high TSS content and TA content compared with the control group. In addition, a higher brightness and lower red transition rates were found in the ACP-treated group. Moreover, the 60 kV ACP treatment significantly inhibited the degradation of chlorophyll, the accumulation of carotenoids, and the activities of chlorophyllase and PAO, and it significantly downregulated the relative expression of *CLH1*, *PPH*, and *RCCR*. Among the three treatment groups, the 60 kV ACP treatment was the appropriate plasma treatment intensity for maintaining high-quality postharvest tomatoes. These results provide some basic data for the future application of ACP to postharvest tomatoes. Additionally, it is necessary to further analyze the mechanism of the effect of ACP on the storage quality of postharvest tomatoes from a variety of perspectives.

## Figures and Tables

**Table 1:** Primer sequences (5′-3′).

Gene Abbreviation	Primer Sequences (5′-3′)	
	Forward Primer	Reverse Primer
*GRAPH*	ACCACAAATTGCCTTGCTCCCTTG	ATCAACGGTCTTCTGAGTGGCTGT
*PAO*	GCATTCCGAAATTGGCTTAGAC	GCTAATCCAGCACTTATAATTGC
*PPH*	GTGTCGAATGAACAATGTACC	CCATTGAGAAGTCATTGATCC
*CLH1*	GGTAGACTTGCTAGTGACCTG	CAAGCTGGCTTGCAACATTCG
*CLH2*	CTCTAAAATTCTCAGCACTCC	GACCATAATCCTTAGCAAGG
*CLH3*	CTCATGTTGGGCCAAATTTG	ACCATAAGTTGCCTTTCCTC
*CLH4*	GCTGAGTTTTTCAACGAGAG	CAGGATCAAGTTTAATAGGAC
*RCCR*	TTTCATACTTGGTAGTTGGGTTCA	GTCCTTTCGCGGAGGTAGAT

**Table 2:** Effects of different intensities of ACP treatment on the color of tomatoes.

Color	Treatment	Storage Time					
		0 Day	7 Day	14 Day	21 Day	28 Day	35 Day
L*	Control	60.10 ± 0.25 ^a^	58.22 ± 0.24 ^b^	55.56 ± 0.23 ^c^	55.40 ± 0.23 ^c^	53.28 ± 0.22 ^d^	51.43 ± 0.21 ^d^
	40 kV	60.10 ± 0.25 ^a^	57.38 ± 0.23 ^c^	56.95 ± 0.23 ^b^	56.08 ± 0.23 ^b^	55.26 ± 0.23 ^b^	55.35 ± 0.23 ^b^
	60 kV	60.10 ± 0.25 ^a^	58.24 ± 0.25 ^a^	57.72 ± 0.24 ^a^	56.23 ± 0.23 ^a^	56.05 ± 0.26 ^a^	55.95 ± 0.23 ^a^
	80 kV	60.10 ± 0.25 ^a^	55.31 ± 0.23 ^d^	54.70 ± 0.22 ^d^	53.54 ± 0.22 ^d^	54.54 ± 0.22 ^c^	54.00 ± 0.22 ^c^
a*	Control	−3.44 ± 0.01 ^a^	−1.57 ± 0.01 ^a^	2.90 ± 0.01 ^a^	8.62 ± 0.02 ^a^	9.88 ± 0.04 ^a^	12.26 ± 0.05 ^a^
	40 kV	−3.44 ± 0.01 ^a^	−2.27 ± 0.02 ^b^	1.89 ± 0.01 ^b^	5.12 ± 0.02 ^b^	8.00 ± 0.03 ^b^	10.54 ± 0.05 ^c^
	60 kV	−3.44 ± 0.01 ^a^	−3.14 ± 0.02 ^b^	−2.77 ± 0.01 ^d^	−1.17 ± 0.01 ^d^	−0.07 ± 0.01 ^d^	3.09 ± 0.02 ^d^
	80 kV	−3.44 ± 0.01 ^a^	−2.85 ± 0.01 ^c^	1.24 ± 0.01 ^c^	4.73 ± 0.02 ^c^	7.03 ± 0.02 ^c^	11.57 ± 0.05 ^b^
b*	Control	19.47 ± 0.08 ^a^	21.55 ± 0.07 ^a^	24.21 ± 0.09 ^b^	20.22 ± 0.09 ^a^	20.30 ± 0.07 ^a^	19.76 ± 0.06 ^b^
	40 kV	19.47 ± 0.08 ^a^	22.75 ± 0.09 ^a^	24.48 ± 0.10 ^a^	20.20 ± 0.08 ^a^	20.44 ± 0.09 ^a^	20.07 ± 0.08 ^a^
	60 kV	19.47 ± 0.08 ^a^	22.66 ± 0.09 ^a^	24.38 ± 0.10 ^ab^	20.32 ± 0.08 ^a^	20.39 ± 0.08 ^a^	19.94 ± 0.08 ^ab^
	80 kV	19.47 ± 0.08 ^a^	22.59 ± 0.08 ^a^	24.26 ± 0.08 ^ab^	20.29 ± 0.07 ^a^	20.32 ± 0.08 ^b^	19.92 ± 0.07 ^ab^

Bars labeled with different small letters (a–d) indicate significant differences among different ACP treatments at the same storage time (*p* ≤ 0.05).

## Data Availability

Data is contained within the article.
